# Insights into the oral health crisis amongst pre-schoolers in Aotearoa/New Zealand: a discourse analysis of parent/caregiver experiences

**DOI:** 10.1186/s12903-020-01173-9

**Published:** 2020-06-30

**Authors:** Michael Roguski, Karen McBride-Henry

**Affiliations:** 1 Kaitiaki Research and Evaluation, 47 Rawhiti Terrace, Kelburn, Wellington, 6012 New Zealand; 2 Health Services Research Centre, Victoria University of Wellington, PO Box 600, Wellington, 6140 New Zealand

**Keywords:** Oral health, Pre-school, Discourse analysis, Public health, Health promotion, Minority populations, Indigenous peoples

## Abstract

**Background:**

The oral health of pre-schoolers is garnering international as a crisis as good oral health is a key precursor to positive health outcomes. Internationally, and within Aotearoa/New Zealand, responses been restricted to those based in a medical model and the commercialisation of oral health. Absent from existing commentary are the lived realities of parents/caregivers beliefs, attitudes and responsiveness, or lack of, to the oral health of pre-schoolers.

**Methods:**

The researcher undertook a discursive analysis of parents/caregivers narratives to understand the barriers to engaging in effective protective behaviours. The 15 focus groups were conducted in urban and rural locations across Aotearoa/New Zealand.

**Results:**

A discursive analysis revealed several pervasive discourses, including ‘second chance’ and ‘enjoyment’ discourses, and systems-related deficits that act as barriers to engaging in good oral healthcare practices.

**Conclusions:**

The analysis demonstrates the benefit of placing the lived experiences of parents/caregivers as central to the development of oral health interventions. There is a need to link oral health data with primary care data and to distribute accurate oral health information to support parents’/caregivers’ decision making. This research reveals several pervasive discourses and systems-related deficits that provide a fertile ground for future public health responsiveness.

## Background

Globally, oral health is a major concern, with oral disease affecting more than 3. 5 billion people [1–3]. Sustained poor oral health is linked to poor health outcomes [2, 4]. These include pain, tooth loss, poor nutritional intake, social isolation and in is one of the leading contributors to the Years Lived with Disability (YLD) [5–7]. Within high-income countries, a highly medicalised approach has become the norm, but without universal coverage marginalised groups are excluded [2]. Equally, in countries with universal coverage, such as in Aotearoa/New Zealand, socio-determinants of health lead to inequitable access to services [2, 8]. This has resulted in experts calling for a complete overhaul of the current system with a focus on maintaining oral health through health promotion efforts [2, 3, 9]. Given that good oral health starts in young children [4] it is surprising that health promotion efforts have been developed based on a medicalised model that is external to the lived experiences of parents/caregivers of pre-schoolers. This has become increasingly important given recent findings that indicate health promotion efforts have failed to assuage a growing crisis of pre-school children. This research was conducted with the aim of exploring the lived experiences of parents/caregivers of pre-schoolers within the context of a high-income country. Research of this nature provides insights into the manner current health service delivery can be re-oriented and assist in identify actionable areas for public health and health promotors.

The prevalence of oral disease is extremely high [1]. Globally, oral disease is viewed as a public health crisis [3] with oral health contributing to a significant disease burden, especially in children [1, 9]. This has resulted in calls for global policy development to prompt collective governmental response to the oral health crisis [1–3, 9].

Good oral health begins in early childhood; however, aggressive tooth decay, or Early Childhood Caries (ECC), which occurs in pre-school children, positions the young person for ill health in later life [2, 10, 11]. Children are at risk of ECC from the time teeth first erupt [12]. The identified risk factors for developing ECC include a complex interplay of dietary, economic, biological and social factors [13–16]. In Aotearoa/New Zealand, ECC is the most common chronic condition amongst children [5]. Those affected at higher rates of the disease are those in low socioeconomic groups, rural populations and particular ethnic groups, namely Māori and Pacific peoples [17–19]. The impact of ECC include the inability to eat, on-going pain, learning and social challenges and poor weight gain [5, 7].

Amongst high-income countries ECC prevalence rates are between one and 12% and up to 70% in low income countries [20]. The most recent published rates in Aotearoa/New Zealand indicate that approximately 50% of all five-year olds have ECC [21], with this rate remaining stable overtime [5].

### Discursive analysis of the issue influencing oral health

Two hegemonic discourses can be identified concerning the issue of oral health: the medicalisation of oral health and the commercialisation of oral health.

The first discourse positions oral health solely within the domain of medics. This comes with a medicalised need response, namely high-income countries invest heavily in cutting edge technology at the expense of accessibility and ‘true’ universal access and indifference to low-cost health promotion strategies [3, 9]. This discourse marginalises those most in need, leaving them with few options to address oral health. Importantly, in privileging the medical model, professional medical responses have been formulated in isolation, and external to, the lived realities of parents/caregivers.

Next, the ‘commercialisation of oral health’ means that marketing, or oral health promotion activities, have been delegated to companies who are financially invested in commodifying their products. This means that factual information about brushing and toothpaste is inaccessible to certain social-demographic population groups and the focus is on the marketing of products and not on the oral health of a population group.

An aggravating factor is the role of food lobbyists who represent companies that produce high-sugar food products. Despite health advocates ongoing efforts to advocate for global action growing concerns about high sugar products has only resulted in a fragmented response, with some countries opting to implement a sugar tax [9, 22].

Rather than understanding the lived realities of parents/caregivers, international studies have generally concentrated on evaluation of programmes designed to improve children’s oral health. These studies have made significant contributions to programme design, highlighting the importance of parents’/caregivers’ desire for culturally-responsive education [19, 23] and parental perceptions and identified ambivalence about the importance of primary teeth [23, 24], a general lack of knowledge about dental services offered [25, 26] and how to correctly brush teeth [25]. Importantly, these studies’ evaluation foci have meant that parent/caregiver perceptions have only been gathered in relation to a particular intervention rather than first developing an in-depth understanding of parent/caregiver lived realities to contribute to a public health response.

Research into the experience of parents/caregivers caring for pre-schoolers oral health is limited within the context of Aotearoa/New Zealand and has focused on oral health knowledge, and related practices, as opposed to beliefs and attitudes that might underpin practice [17, 19, 27, 28]. Further, extant literature has overly relied on surveys to examine parent/caregiver knowledge and practice (see for example, Chan et al., 2002; Mani et al., 2012; Rothnie et al., 2012). This type of analysis, although helpful in understanding population trends, fails to account for the richness or depth of lived realities of parents/caregivers when caring for their pre-schooler’s oral health. For instance, Schluter and colleagues (2007) interviewed 1048 mothers of four-year old children living in Aotearoa/New Zealand, using a face-to-face survey to gather data. The findings revealed that overall Pacific children had poor oral health practices, with 26% of the group having not yet seen a dental professional and the reported rate of tooth brushing was low when compared with ideal practice. Next, findings from a survey of Māori mothers’ understandings of oral health highlighted the important role that a Māori mother in nurturing her children and identified the need these mothers to have meaningful oral health information. Another study interviewed six parents to explore knowledge of this topic. The research indicates that parents/caregivers did not have enough information to provide care for the oral health of their children [27]. Further, this research highlighted parent/caregiver, child and family level influences on oral health for preschool children and explored access to services, revealing myriad issues at play and directly impacting oral health practices.

Within the context of a growing oral crisis there is a need to extend our current understanding of possible contributing factors away from a solely medicalised focus to understanding the lived realities of parents/caregivers caring for their pre-school children’s oral health care. This research aimed to explore these lived realities and understand possible discourses that can be harnessed to contribute to improved health interventions.

## Methods

A qualitative approach was employed to explore the lived realities of parents/caregivers in relation to caring for their preschool child’s oral health. A discourse analysis methodology underpinned the philosophical approach. Researchers employed discourse analysis as a methodology to focus on the language used by participants, but concentrating on concepts versus individual ideas, as well as, the situation in which language is used [29]. This methodological approach emphasises the meanings that are fashioned from the social process that occur through the dialogical process [30].

This study was reviewed and approved by the New Zealand Ethics Committee prior to the research commencing (New Zealand Ethics Committee ID 2015#23).

Recruitment occurred through purposive sampling methods, with participants being contacted through different community leaders who were aware of the aims and intent of the study. The eligibility requirements included: being 16 years or older; being a parent/caregiver to one or more pre-school aged child (six months- four years of age); the adult responsible for oral health of the pre-school child. The purposive sampling method was employed because of a desire to recruit people within the lower socioeconomic group to participate in the research.

Fifteen focus groups, with a total of 107 participants, were facilitated across five different geographic locations: Auckland, Christchurch, Gisborne, the Wellington Region, Whangārei and a small village in the Far North of the North Island of Aotearoa/New Zealand. Diversity amongst participant groups was sought by organising the focus groups according to ethnicity and through the varied geographical locations (see Table 1). This resulted in six focus groups being conducted with whānau Māori (extended family) (in Wellington, Tairawhiti, Christchurch and Northland) and Pacifica (in Wellington, Northland, Auckland), plus a total of three focus groups with New Zealand European (Wellington and Christchurch). A koha (gift) of $50.00NZD was given to each participant.

**Table 1:** Focus group characteristics by location

Locality	Geographic Location	Number of focus groups in each region	Total number of participants in focus groups	Ethnicity	Median household incom /year	Deprivation for location
**Rural**	Small village in the Far North	1	13	Māori	$4999–$20,000	High
	Gisborne	1	6	Māori	<$60,000	High
**Urban**	Whangārei	1	6	Māori	$40,000-$100,000	High
		2	12	Pacific Peoples	$21,000-$40,000	High
	Wellington region	2	12	Māori	$4999-$80,000	Medium/ High
		2	12	Pacific Peoples	<$60,000	High
		2	12	NZ European	>$100,000	Low
	Auckland	3	23	Pacific Peoples	<$60,000	High
	Christchurch	1	6	Māori	$4999-$20,000	High
		1	5	NZ European	>$100,000	Low

The average focus group size was seven parents/caregivers, although total numbers ranged between five to 13 participants. Two of the focus groups had local kaumatua (older person of status) attend and they were welcomed to join in because of the cultural contribution they were able to make to the research. Verbal and written consent was obtained from the participants. The focus groups were between one and two hours in duration, and each focus group was audio recorded.

Focus groups were semi-structured. They began with the facilitator asking a series of short true/false questions, adapted from Ministry of Health guidelines [31], to introduce the topic and trigger further discussion amongst participants. There was a total of eight general oral health questions covering a variety of oral health topics, such as when to enrol a child in an oral health service and whether it is acceptable for your child to share toothbrushes.

Most of the participants identified as female (*n* = 82, 76.6%) and ages ranged from 18 to 65 years; the average age of the participants was 31 years. Most of the participants reported having between one and three children in their households (*n* = 75, 70.1%), while 18 (16.8%) had between four and five children and 14 (13.1%) had six more children in the household. Information about household income was gathered with the average reported income being $40,000NZD per year; participants indicated their income ranged from $5000 to $249,000NZD. Of note, approximately one third of participants reported a household income of between $4999 and $20,000NZD. This is significant given that Aotearoa/New Zealand has an average household income before-tax of $105,719NZD [32]. Participant ethnicities included Māori (*n* = 51, 47.7%), Pacific peoples (*n* = 35, 32.7%) and New Zealand European (*n* = 21, 19.6%).

A discourse approach was employed to analyse focus group transcripts. Emerging themes focused on the presence and interplay of ubiquitous discourses. The process involved: attending to the participants’ context; focusing on the language employed by the participants to describe their experiences; and centring on the emergent discursive themes. Throughout the research fieldwork emerging discourses were constantly re-analysed and the focus group conversations were used to build and extend knowledge during subsequent focus groups. This meant that emergent discourses could be tested and constructed leading to the creation of the analysis framework. During the fieldwork, the transcripts and fieldnotes were constantly reviewed and discourses were identified and tested with participants within the context of the focus groups. This process of discursive analysis also affords the research the opportunity to investigate the meaning embedded in these emerging discourses. Participants’ narratives were drawn upon to illustrate the emergent discourses.

## Results

### Participant contexts

Based on participant narratives, two distinct groups were identified which differed according to knowledge of pre-school oral health. Results of an oral health quiz indicated that one group was highly motivated to seek out information about oral health and readily accessed social networks and health professionals to garner dental and health-related information. Of note, this group were more likely to have older children, which had afforded them the opportunity to have learnt about dental care through previous experience. Significantly, a high degree of social connectedness provided this cohort with extensive opportunities to maintain a high degree of oral-related agency.

Those whose quiz scored indicated a low level of knowledge about oral health for pre-schoolers differed from former cohort across most elements. The cohort was denoted by a lack of agency and low levels of social connectedness, which acted to compromise the cohort’s opportunity to share and gain knowledge of oral health for pre-schoolers. Furthermore, the cohort tended to be younger, typically 20-years of age and under, possessing limited knowledge of oral health care, nutrition and were unaware of the importance of oral health and general wellbeing. The cohort was also more likely to be socio-economically disadvantaged. Finally, associated with low levels of oral health literacy, there was a distinct lack of trust of the State and health professionals with preference given to a trusted individual within their isolated social circles. This undermined organised efforts to improve health literacy as this cohort did not trust the information provided by health professionals or the State.

### Motivations underpinning oral health care for pre-schoolers

Most participants placed a high degree of importance on their child’s oral health care. The following three primary motivations were identified: physical appearance, prevention and maintaining family traditions.

A primary motivator for parents/caregivers to ensure their child had regular access to oral health care was the importance of physical appearance. Initially the value placed on oral health was portrayed as a cosmetic consideration, such as “looking good” or “having a nice smile”. However, the cosmetic focus was later qualified through narratives that highlighted a desire for their children not to be socially marginalised or shamed. Such motivations were founded in the parent/caregiver childhood experiences of either being bullied or experiencing social ostracism because of having “bad” teeth and/or poor oral hygiene.

*When I was at school kids with bad teeth would get picked on. You know, made fun of. I don’t want my kids to feel that type of shame.*

Prevention as a motivation was common across participants and was discussed as existing in differing degrees. Initially, parents/caregivers discussed how they wished to avoid potential painful dental procedures for their children. These narratives were also borne out of the parent/caregiver’s personal childhood experiences.

*My girl had some cavities and getting the fillings was really painful for her. Since then I make sure my children brush twice a day. I don’t want to see my kids in pain.*

*When I was a kid no one made me brush my teeth. I had terrible teeth. They were yellow from a young age and I had a lot of teeth pulled. So it’s really important to me that my kids don’t go through that.*

In addition, motivations associated with preventing poor oral health centred on the need to reduce adulthood-related complications. Participants strongly asserted that childhood provides an optimal opportunity to embed good oral health practices, thereby avoiding later-in-life dental-related financial costs.

*Dentists are so expensive. I didn’t look after my teeth when I was younger and it has been really expensive for me. So I try and make sure my kids look after their teeth and it becomes a habit. That way they won’t have to go through all the expenses I have had to face.*

Next, a significant motivation for engaging in preventative oral health care arose from a desire to avoid peer-based judgment. Prevention, therefore, became a mechanism by which parents/caregivers could avoid being judged. Of note, this was a particular issue for New Zealand European participants.

*I guess a big motivator for me is that I see my kids teeth as reflection of my parenting. If they have bad teeth I feel as though people would judge me as a bad parent.*

The final motivation was deference to historical family practices: a motivation associated with a pride in one’s families’ history of good oral health.

*My family, like my parents and grandparents, have always had good teeth. It’s something the family has always talked about. If my kids have bad teeth then it feels like I would be breaking a tradition.*

### Understanding barriers to effective oral health behaviour

Two forms of barriers arose from the focus groups: discursive barriers and systemic barriers. Discursive barriers reflected beliefs, attitudes, knowledge and familial systems, generally involving the extended family structure, that impacted on the pre-schooler’s oral hygiene. Systemic barriers included factors external to the familial unit that impacted on engagement and service receipt.

#### Discursive barriers

Parents/caregivers who communicated a lack of adherence to pre-school oral hygiene commonly framed their practices within two primary discourses. Firstly, the need for pre-school oral care was minimised by a belief that baby teeth are temporary and adult teeth provides an opportunity to access oral health care later in life. This *second chance* discourse represented a lack of knowledge about the need for preventative oral health care during pre-school years.

*I don’t make my kids brush because they only have their baby teeth. Those teeth aren’t important really. The adult teeth are much more important. When they lose their baby teeth I will make sure they brush regularly.*

Linked to the *second chance* discourse, a minority of parents/caregivers articulated that they were not at all concerned about their child’s pre-school oral health practices.

*It takes so much time and I am just lazy. Also, at the heart of it I guess I just don’t think it is that important.*

Parents/caregivers described their role as supervisory, at best, with parents/caregivers intervening when there was an obvious issue with their child’s oral health arose. Parents/caregivers who held this belief discussed how they ceased providing any direct involvement in their child’s oral health from the age of 2 years and older.

*I only make them clean their teeth when their breath smells bad.*

The most pervasive discursive barrier to pre-school oral hygiene was *familial*-*enjoyment*. Oral health care was designated as the parents’/caregivers’ responsibility. As such, extended family members, such as grandparents, aunties and uncles, claimed the right to “*enjoy*” their pre-school children – enjoyment occurring at the expense of oral health considerations. As a consequence, a high degree of frustration, powerlessness and anger was registered amongst parents/caregivers. Grandparents were often the source parent/caregiver frustration, who reportedly ignored good oral health practices so they could reward and enjoy their grandchildren.

*My job is to enjoy my grandchildren. It is the parent’s job to look after their teeth. My job is to have fun with them. They love coming to my house. They run in and know that I will have sweets and cakes for them … I love spoiling them.*

Family members’ non-adherence to oral health care resulted in extreme consequences, such as tooth extractions and adolescent dental problems. In addition, interference in oral health routines resulted in heightened tension for parents/caregivers, who often found it exceedingly difficult to reinstate routines when the child was returned to the parent/caregiver’s care. Many participants reported that they either lacked confidence or strategies to challenge family members; often citing poor outcomes from previous attempts, such as strained relationships and no changes to the family member’s behaviour.

*It’s actually become a really big issue for me. I feel as though I am trying my best to look after my kids and then my parents just override everything that I have put in place. I’ve tried all kinds of things. Like sitting down with my parents and trying to explain why I don’t want my kids to have sugar. They [parents] just thought I was being unreasonable and that I was depriving my children and they just carried on doing what they wanted. More recently I have said that the kids can’t stay at their house unless they respect my wishes. That hasn’t gone down well [laughter].*

#### Systemic barriers

A number of barriers were identified as negatively impacting on pre-school children’s timely access, and sustained engagement in, oral health care. These included a lack of awareness of national health recommendations, systemic inconsistencies, access-related service barriers, and the commercialisation of dental health products. The latter was reported as placing lower income families under considerable financial duress.

The majority of participants had no knowledge about the national health recommendation that a child should be registered with a local oral health provider from birth. Most Pacifica reported knowing the guideline whereas most Māori and New Zealand European were unaware of such recommendations.

Others reported receiving contradictory messages from health professionals, such as the age at which a child should have their first and follow-up appointments. In addition, confusion about the enrolment process was discussed. Some described completing the enrolment application up to 1.5 years before the focus group but had not received an acknowledgement from the provider. Others described confusion over who was responsible for enrolling children, not realising that it was the role of primary care providers. In some cases, the visiting nurse service’s made the referral after the first home visit while others described primary health providers as having no obvious referral role, placing the onus on parents/caregivers to make oral health appointments. Overall, there was a high degree of agreement that the process of enrolment in an oral health service was convoluted and not easy to navigate, which meant that pre-school children were often delayed in accessing the dental service or missed their first appointment completely.

Participants also identified sustained engagement with oral healthcare services as another challenging issue. This issue was emphasised when a family relocated, as often no forwarding information was provided to oral health care providers. Of note, there appeared to be a lack of administrative consistency surrounding the health providers’ provision of new addresses to relevant district health boards.

In addition, some participants reported difficulties accessing oral health services. Such difficulties included accessing and wait times for oral health services, within prescribed hours, when the service was a considerable distance from day care facilities. This was especially an issue noted by in more provincial areas.

*Up here [provincial Aotearoa New Zealand] it takes so long to get an appointment. It took me 2.5 months last time.*

*I had to wait ages for an appointment and then I had to drive two hours to get there; four hours in total.*

Additional barriers to pre-school oral health were associated with the commercialisation of dental health products. Most significantly, participants described considerable confusion about the use of fluoridated toothpastes as they were able to buy unfluoridated toothpastes at commercial outlets. Similarly, age-branded toothpastes were perceived to be specially formulated for particular age groups and were perceived as the healthiest pre-school child-aged option.

At the heart of the misinformation associated with age-branded toothpastes, was an issue of affordability. Affordability was identified as a systemic issue that placed consider duress on families from low income households. These families described having made financial sacrifices in order to buy child-age-branded toothpastes as participants reported that branding indicated that child-aged branded toothpastes were specially formulated. Further, participants described a belief, reinforced by marketing, that cheaper toothpastes were less effective than more expensive brands. Consequently, some participants had chosen to purchase more expensive toothpastes, accepting the financial burden that came with this, because of a distrust of cheaper options.

The availability of unfluoridated and age-branded toothpastes were perceived to have communicated a message of government endorsement of the available commercial products by parents/caregivers. As part of the focus group discussion participants were informed about government health-related guides and that age-branded toothpastes had no additional benefit than a generic household toothpaste. Importantly, participants communicated considerable anger and frustration upon discovering their purchasing behaviour had been influenced by marketing, an unnecessary financial expense given health recommendations that one toothpaste can be used for the entire family.

Within a public health context, it is pertinent that the availability of health-related commercial products are assumed to be endorsed by government, an endorsement that validated the additional expense.

*I just don’t understand it. Why are there low fluoride toothpastes in supermarkets if the government is saying that we should use the fluoride ones? It’s really confusing and is sending mixed messages.*

*When you are in the supermarket and you see all these different toothpastes that say something like, “For children aged zero to three years or for kids seven years and up” it gives the impression that is approved by the government. So you buy it because you think it is the best thing for your kids.*

The following quote echoes many participants’ experiences, the grandmother who offered the narrative had an annual income of around $18,000 NZD and was responsible for the care of three children.

*I am a grandparent and I raise three of my grandchildren fulltime. So I am on a benefit and I have little in the way of money. The only things I buy are necessities. So I am sitting here [in the foucs group] and I am relaly angry to find out that there is no need to buy children’s toothpaste, that [it] is just marketing. I really thought I was doing the best for my moko (grandchildren) but it was money I could have put to better use.*

## Discussion

The dominant medical model has developed oral health strategies in isolation; external to a demonstrated understanding of parents/caregivers lived realities. Further, the continued reliance on a reductionist paradigm has effectively nullified the parents’/caregivers’ voices. Given a growing acknowledgement that preschool oral health care is a burgeoning crisis, opportunities exist to alter extant approaches. The authors of this current study have attempted to disrupt reductionist convention by gathering the lived realities of parents/caregivers and have identified a number of discourses that can be harnessed to contribute to improved health responses for the preschool population.

Two primary discourses, embedded within family units, were identified. The prevalence of a ‘second chance’ discourse provides a strong indication for the need for improved public health messaging to combat the minimisation of pre-school oral health because of a belief that the first set of teeth do not matter and any dental issues experienced will be remedied by arrival of adult teeth; this issue has also been highlighted by other researchers [23, 27]. Similarly, participants’ narratives strongly indicate a need for messaging surrounding the predominance of an ‘enjoyment discourse’ for the wider family unit.

The enjoyment discourse is founded on the premise that a child’s oral hygiene and dental care is the sole responsibility of parents/caregivers. As such, preventative dental care is not considered to be the responsibility of the extended family. Such positioning, however, counters much of the holistic understanding fundamental to Māori and Pacific family units, whereby the centrality of the whānau, and not the individual, is reflective of epistemological worldviews. Given the cultural importance of familial cohesiveness and shared responsibility, we further that public health messaging should entail a relatively practical shift whereby targeted messages are developed to include the wider social network of pre-schoolers’ families; thereby, ensuring all family members recognise the importance of oral health for pre-schoolers and the role that diet plays in supporting good oral health. This strategy is informed by a variety previous successful health promotion campaigns that have utilised the centrality of family units as a means of furthering health responsiveness. Such models include smoke free initiatives, cervical smears and reducing hazardous alcohol intake.

Next, parents/caregivers’ struggled to ensure their pre-schooler could access and maintain their involvement in preschool oral health services because of a number of systemic barriers. In part, informational barriers can be rectified through improved public health messaging, for instance there is a need increase awareness of Ministry of Health recommendations involving dental hygiene and the provision of pre-school dental care. The latter provision, however, is contingent on parents/guardians enrolling the child in an appropriate service. There is an evident need for guidance around this process. Importantly, however, the onus of responsibility sits equally with the health professionals and the State. Training is required for health professionals to minimise the conflicting messaging experienced by parents/guardians. The delivery of dental health information also needs to be addressed to ensure that dental health, as with other health considerations, is not lost in what one participant described as “an onslaught of information”.

It is noteworthy, a noticeable degree of commonality existed across the discourses. However, those who identified as Māori, to a lesser extent Pacific peoples, and those from low-socioeconomic areas, experienced significant challenges when navigating the various barriers arising out of these discourses. This included a lack of personal agency to be able to access oral health care initially and remain engaged in a service, in an ongoing capacity. Compromised agency reinforces the negative health impacts experienced by those embedded in communities of high economic deprivation. In contrast, those who identified as New Zealand European, or had a higher level of income; demonstrated a higher degree of agency, and therefore, had the ability to overcome the systemic barriers they encountered.

The State’s role in dental hygiene and care was raised on three levels. Firstly, the State has a role in removing confusion and administrative inefficiencies that result in an administrative failure to either acknowledge the child’s enrolment or having failed to enrol the child despite the parent/caregiver’s best efforts. As such, effective strategies for enrolling infants need to be considered by policy makers and health system designers. Because oral health is considered to be a separate service, residing in isolation from other primary care providers, we recommend that oral health be viewed in a less reductionist manner. Next, the State has a role in ensuring the child’s sustained engagement with oral health care professionals. Protracted waiting times and access-related barriers need to be reviewed and sufficient resourcing allocated. This is most notably an issue in provincial communities. Sustained engagement was also identified as a significant issue for relocating families, as moving geographic locations resulted in a lack of follow-up with the preschool oral health service. Parents/caregivers did not know how to access the oral health service in their new locations nor how to ensure information was forwarded to a new provider. We further that disengagement could be remedied, at least in part, with the linking of the child’s primary care information to her/his oral health data.

Finally, the State was regarded as complicit in the prevalence of confused messaging associated with the commercialisation of dental healthcare products. These included the availability of unfluoridated toothpastes at commercial outlets and the prevalence of age-branded toothpastes, which were perceived to be specially formulated for particular age groups. The significance of participants’ unease about the State’s perceived endorsement of commercial products needs to be appreciated in light of the fact that the majority of participants are from low income households. Within this context, families had made financial sacrifices to purchase products, such as age-branded toothpastes, because they believed such branding would only be permitted with the State’s endorsement. Similarly, the availability of unfluoridated toothpaste was equally regarded as something permitted by the State, and only added to the confusion of whether, or not, fluoridated was a healthy option. Given the current crisis of dental hygiene and care facing our pre-school children, there is an urgent need for the State to align its health recommendations with available commercial products. We assert that the most practical approach would be for approved products to carry appropriate branding while simultaneously promoting evidenced-based messaging to guide product selection.

## Conclusion

This research has examined existent discourses available on the topic of pre-schooler’s oral health; two hegemonic discourses were identified within the literature - the medicalisation of oral health and the commercialisation of oral health promotion. These discourses have shaped societal understanding of pre-schoolers’ oral health while excluding the voice of parents/caregivers; silencing their understandings and ability to influence public health initiatives to improve the oral health of their pre-schoolers. This research has explored discourses that emerged from 15 focus groups conducted with parents/caregivers of preschool children, many of whom come from Indigenous, minority ethnicities and areas of high socio-economic deprivation. The analysis highlights participants' confusion surrounding  correct oral health practices, challenges negotiating with wider whānau and family, and barriers to accessing oral health care. The findings demonstrate that parents’/caregivers’ are able to identify modifiable issues that negatively impact upon their pre-schoolers’ oral health. These findings were more pronounced for those in areas of high deprivation and amongst the Indigenous population; highlighting an issue of decreased agency. This research also highlights the need to have parents/caregivers input into public health initiatives and ways in which service delivery can be enhanced accordingly. The onus rests with the State to address the concerns around oral health access for pre-schoolers and to engage in the extensive distribution of accurate oral health information.

## Supplementary information

**Additional file 1.** Focus group schedule. Research on preschool oral health. Focus group schedule that was used in the fieldwork for this research.

## Data Availability

Data sharing not applicable to this article as no datasets were generated or analysed during the current study. The narratives collected during this research are unavailable because of ethical restrictions relating to being able to identify participants and consent processes that prevent the researchers from sharing them.

## Abbreviations

YLD: Years Lived with Disability
ECC: Early Childhood Caries
Māori: Person Indigenous to Aotearoa/New Zealand
Pacifica: Person who has ethnic connection to a Pacific Island nation
European: Person who has ethnic connection to a European nation
Whānau: The term used to describe wider family structures amongst Māori
