# Supplementary material for: Insights into the oral health crisis amongst pre-schoolers in Aotearoa/New Zealand: a discourse analysis of parent/caregiver experiences
Source: BMC Oral Health. 2020 Jun 30;20:182. doi: 10.1186/s12903-020-01173-9 (PMC7325262; doi:10.1186/s12903-020-01173-9)
Supplement: Supplementary file 1 — Additional file 1. Focus group schedule. Research on preschool oral health. Focus group schedule that was used in the fieldwork for this research. [file 12903_2020_1173_MOESM1_ESM.docx]

#### Research on preschool oral health

##### Focus group structure and question prompts

1. **Introductions and warm up (5 minutes)**

- thanks for coming, group to last approximately 2 hours.
- confidentiality/informed consent and audio-recording.
- explain the purpose of the focus groups
- explain process - group discussion, no wrong answers.
- ground rules - give it a go, respect each other, everyone contributes.
- housekeeping - toilets, fire exits, food etc.
- participant to introduce themselves

1. **Quiz (10 minutes)**

Start the focus group with a group quiz based around the Ministry of Health’s current messages. The aim of the quiz is to:

- determine the groups’ level of knowledge about preschool oral health
- provide a basis for discussion.

| **Quiz Questions**  **(Italics are the actual questions. Normal font are the MOH guidelines)** | **Yes (total)** | **No (total)** |
| --- | --- | --- |
| 1. *It is recommended that you enrol your child at birth in your local Community Oral Service.*   Regular dental visits are vital from an early age. Your child is entitled to free check-ups and basic oral health services from your local Community Oral Health Service. Enroll your child at birth. |  |  |
| 1. *You should not start brushing your baby’s teeth as soon as they appear through the gums*   Start brushing baby’s teeth as soon as they appear through the gums, usually around 6 months of age |  |  |
| 1. *Low fluoride baby or junior toothpastes are recommended*   Use regular strength fluoride toothpaste for all family members – low fluoride baby or junior toothpastes are not recommended-– there can be one toothpaste for your whole household. |  |  |
| 1. *You should always ask your child to rinse after brushing their teeth.*   Brush and spit. Don’t rinse. Spitting instead of rinsing keeps the toothpaste working long after brushing and helps to better prevent tooth decay |  |  |
| 1. *You will need to supervise and help with your child’s tooth brushing until they are about 8 years old.*   You’ll need to supervise and help with your child’s toothbrushing until they are about 8 years old. You need to supervise your child to ensure they are not eating toothpaste and you’ll need to put on the toothpaste, because small children will nearly always put on too much toothpaste. Using too much toothpaste and eating it can cause white speckles on your child’s adult teeth. Once your child can control a pencil and begins to write (when they are about 5 years old), they should be able to brush their own teeth. |  |  |
| 1. *Your child will learn most about how to clean their teeth by copying you.*   Make sure you look after your own teeth well. Your child will learn most about how to clean their teeth by copying you. |  |  |
| 1. *It is okay for your children to share toothbrushes.*   Make sure everyone in the household has their own toothbrush. Your child can catch bacteria from you that cause tooth decay – for example, from sharing spoons or your toothbrush. |  |  |
| 1. *Juice is the best drink for your child’s teeth.*   Water and milk are the best drinks for teeth. |  |  |

1. **Motivations (20 minutes)**

What are the motivations you have for your child to have good oral health?

- how important is it for you that your child brushes their teeth regularly (ie. twice a day everyday)?
- can you tell me why?
- what do you want to achieve?
- is this a priority? If yes, why?
- if not a priority, why?

1. **Barriers of engaging in effective protective behaviour (25 minutes)**

I’m really interested in knowing how difficult is it for your child to have good oral health.

- what needs to happen to ensure your child has good oral health?
- motivation?
- cost?
- knowledge?
- child’s non-compliance?
- work-life balance?
- knowledge of child development?
- access to information?
- not part of a social or other type of group where this type of information is readily shared or available?

**It might be easier if we look at problems you encountered when your child was at different ages?**

**1 Year Old**

**Firstly, on a scale of drama, where 1 represents no problems with brushing and oral care and 10 represents extreme resistance, tantrums and refusal, where would you rank your child when they were 1 year old?**

- Write down each participants ranking. This will used to generate mean responses later.
- Paraphrase how many children had problems and how many children had no problems. Tell participants that you are going to explore both issues.

**What were some of the problems or barriers you encountered when your child was 1? (If** possible write responses against the drama ranking scale)

**What were some of the things that helped your child over come these issues when they were 1 years old?**

**Some of you said that your child had no problems at 1.**

- Can you tell us more?
- Why do you think you had no problems (If possible write responses against the drama ranking scale)

**2 Years Old**

**On a scale of drama, where 1 represents no problems with brushing and oral care and 10 represents extreme resistance, tantrums and refusal, where would you rank your child when they were 2 years old?**

- Write down each participants ranking. This will used to generate mean responses later.
- Paraphrase how many children had problems and how many children had no problems. Tell participants that you are going to explore both issues.

**What were some of the problems or barriers you encountered when your child was 2? (If** possible write responses against the drama ranking scale)

**What were some of the things that helped your child over come these issues when they were 2 years old?**

**Some of you said that your child had no problems at 2.**

- Can you tell us more?
- Why do you think you had no problems (If possible write responses against the drama ranking scale)

**3 Years Old**

**On a scale of drama, where 1 represents no problems with brushing and oral care and 10 represents extreme resistance, tantrums and refusal, where would you rank your child when they were 2 years old?**

- Write down each participants ranking. This will used to generate mean responses later.
- Paraphrase how many children had problems and how many children had no problems. Tell participants that you are going to explore both issues.

**What were some of the problems or barriers you encountered when your child was 3? (If** possible write responses against the drama ranking scale)

**What were some of the things that helped your child over come these issues when they were 3 years old?**

**Some of you said that your child had no problems at 3.**

- Can you tell us more?
- Why do you think you had no problems (If possible write responses against the drama ranking scale)

**4 Years Old**

**On a scale of drama, where 1 represents no problems with brushing and oral care and 10 represents extreme resistance, tantrums and refusal, where would you rank your child when they were 2 years old?**

- Write down each participants ranking. This will used to generate mean responses later.
- Paraphrase how many children had problems and how many children had no problems. Tell participants that you are going to explore both issues.

**What were some of the problems or barriers you encountered when your child was 4?** (If possible write responses against the drama ranking scale)

**What were some of the things that helped your child over come these issues when they were 4 years old?**

**Some of you said that your child had no problems at 4.**

- Can you tell us more?
- Why do you think you had no problems (If possible write responses against the drama ranking scale)

1. **Cost**

**To what extent is cost a barrier?**

- toothbrushes
- toothpaste
- other.

Just to clarify, where would you put yourself on a 10-point scale? **Where 1 represents no problems with cost and 10 represents cost as a major problem.**

1. **Knowledge and support around your child’s oral health (30 minutes)**

- where did/do you learn about preschool oral health, who was the knowledge giver?
- who do you go to for knowledge and support?
- who would you go to for knowledge and support?
- what are some of the things your have learnt recently that you wish you knew earlier on?
- what are some of the barriers to learning about child oral you have encountered?

(for the answers provided by a group do a frequency count beside each response e.g. Facebook mothers’ Group 5 / 6)

1. **Making pre-school oral health care easier (20 minutes)**

**What would make looking after your pre-schooler’s oral health care easier?**

- where would you most likely like to receive information about pre-school oral health care?
- what support do you need?

**THANKS AND CLOSE**
